# Periconceptional Counselling in Women with Autoimmune Inflammatory Rheumatic Diseases

**DOI:** 10.3390/jcm13092483

**Published:** 2024-04-24

**Authors:** Klara Rosta, Julia Binder, Valerie Kuczwara, Mira Horvath, Florian Heinzl, Christina Hörhager, Daniel Mayrhofer, Peter Mandl, Ruth Fritsch-Stork, Johannes Ott, Antonia Mazzucato-Puchner

**Affiliations:** 1 Division of Gynecological Endocrinology and Reproductive Medicine, Department of Obstetrics and Gynecology, Medical University of Vienna, 1090 Vienna, Austria; 2 Division of Obstetrics and Fetomaternal Medicine, Department of Obstetrics and Gynecology, Medical University of Vienna, 1090 Vienna, Austria; 3 Division of Rheumatology, Department of Internal Medicine III, Medical University of Vienna, 1090 Vienna, Austria; 4 Health Care Centre Mariahilf, ÖGK and Rheumatology Department, Sigmund Freud Private University, 1060 Vienna, Austria

**Keywords:** rheumatic disease, pre-pregnancy counselling, immunomodulatory medications, obstetric risk, disease flare, obstetric outcome

## Abstract

Systemic autoimmune rheumatic diseases (SARDs) in pregnancy represent a complex challenge for both patients and healthcare providers. Timely preparation for pregnancy enables adequate disease control, thereby reducing the risk of disease flare and pregnancy complications. Interdisciplinary care starting from the pre-pregnancy period throughout pregnancy and during breastfeeding ensures better fetal and maternal outcomes. This review provides a comprehensive guide to pre-pregnancy counselling in SARDs, an overview of medication management strategies tailored to pregnancy, disease activity and pregnancy monitoring in patients, and the promotion of shared decision making between healthcare providers and patients. Guidelines from international organizations were selected to provide a basis for this review and guidance through the quintessential discussion points of care.

## 1. Introduction

**Figure 1:**
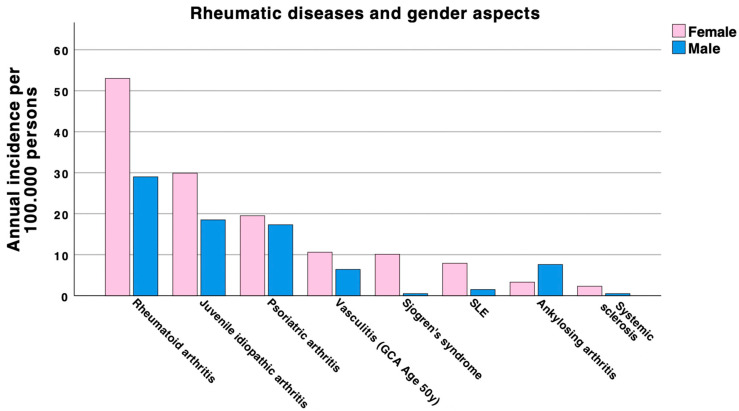
Gender bias of systemic autoimmune rheumatic diseases. Legend: SARDs manifest more often in women. This figure compares the annual incidence of the specific rheumatic disease between male and female persons. Incidences are based on the following literature data: [1,2,3,4,5,6,7,8]. Abbreviations: SLE = systemic lupus erythematosus; GCA = Giant Cell Arteritis.

Systemic autoimmune rheumatic diseases (SARDs) in pregnancy represent a complex challenge for both patients and healthcare providers. These conditions encompass a range of autoimmune disorders, including inflammatory joint disease (IJD), and connective tissue disease (CTD). While they affect patients of all ages and genders, SARDs manifest more often in women, commonly affecting them during childbearing age (Figure 1), making peri-pregnancy counselling and pregnancy management a crucial aspect of care.

**Figure 2:**
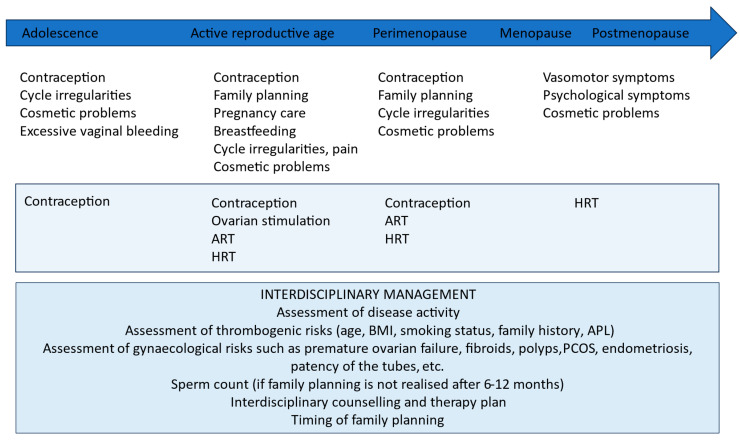
Counselling of women with SARDs during their reproductive lifespan. Legend: In the course of a woman’s reproductive lifespan, distinct challenges may arise as she ages. Different stages, spanning from puberty through active reproductive years to perimenopause and menopause, present varied issues. These challenges encompass the requirement for reliable contraception, pregnancy planning, addressing infertility, managing menstrual irregularities, tackling cosmetic concerns, and alleviating symptoms associated with menopause. The management of these issues in patients with SARDs requires careful evaluation and interdisciplinary approaches. ART: artificial reproductive techniques, HRT: hormone replacement therapy, BMI: body mass index, APL: antiphospholipid antibodies, PCOS: polycystic ovary syndrome, SARD: systemic autoimmune rheumatic disease.

The management of women with SARDs poses challenges to both rheumatologists and gynecologists, highlighting the need for interdisciplinary care covering woman’s reproductive lifespan, starting with counselling about possible contraceptive methods, through reproductive challenges and pregnancy, breastfeeding, and including the periods of perimenopause and menopause (Figure 2).

During pregnancy, patients with SARDs face the risk of disease flares, which can lead to increased pain, disability, concomitant organ damage, and potential pregnancy complications, such as preterm birth, fetal growth restriction (FGR), preeclampsia, stillbirth, or other disease-specific complications which may harm the fetus, such as neonatal lupus [9].

Additionally, the use of medications to control disease activity must be carefully balanced to minimize risks to the developing fetus. The withdrawal of medication due to pregnancy often lead to disease flare, endangering the mother as well as triggering multiple obstetric risks [10,11]. 

Multidisciplinary collaboration between rheumatologists, obstetricians, obstetric physicians, neonatologists, pediatricians, and other specialists, including hematologists, embryologists, nephrologists, dermatologists, radiologists, and reproductive medicine specialists is often necessary to achieve the best outcomes. Furthermore, navigating the complexities of medication safety during pregnancy and monitoring disease activity and obstetric outcomes in a situation of rapidly changing guidelines and still scarce but constantly increasing evidence about medication use in pregnancy is challenging [12,13,14].

This overview describes the intricate challenges of caring for patients with SARDs before and during pregnancy. We will also address the obstacles, which prevent us from attaining better data on medication safety in pregnancies, to draw attention to its importance to researchers and policy makers. Understanding these complexities is essential for delivering comprehensive, patient-centered care to pregnant individuals with rheumatic diseases, and through this, improving the health of both the mother and the newborn.

### Scope of the Review

This review focuses on the elements of pre-pregnancy counselling, medication management strategies tailored to pregnant individuals, disease activity monitoring throughout pregnancy, pregnancy monitoring in rheumatic disease, and the promotion of shared decision making between healthcare providers and patients. We seek to provide a comprehensive understanding of the recommendations and approaches to counselling, which ultimately guide clinicians and healthcare practitioners in delivering optimal care to pregnant individuals with SARDs. 

Relevant guidelines are offered by three key organizations: the European League Against Rheumatism (EULAR) [14], the British Society for Rheumatology (BSR) [13], and the American College of Rheumatology (ACR) [12]. These guidelines are discussed and reflected upon in detail. Differences between the guidelines will also be addressed—these can be partly explained by the different dates of publication and/or a divergent focus. Note that the EULAR points to consider were developed in 2016 and are currently being updated.

## 2. Reproduction and Rheumatic Diseases

### 2.1. Epidemiology

Although SARDs are rare conditions, they collectively impact a significant portion of the population. For instance, rheumatoid arthritis (RA) affects approximately 1% of the global population, with women being two to three times more likely to develop the condition [1]. Similarly, systemic lupus erythematosus (SLE) primarily affects individuals of childbearing age, with an estimated global incidence in women of 8.82 (2.4 to 25.99) per 100,000 person-years [15] and a female-to-male ratio of about 9:1. The prevalence of SLE also varies by region and ethnicity but is generally estimated to be 43.7 per 100,000 persons, ranging from 15.87 to 108.92 per 100,000 persons and 28.61 to 196.33 cases per 100,000 women [6,15]. This underscores the need for comprehensive guidelines and strategies to manage these diseases during pregnancy (also see Figure 1).

### 2.2. Challenges and Complications in Reproductive Age

#### 2.2.1. Fertility in SARDs

Patients with rheumatic diseases have fewer children than healthy individuals. Personal choices, infrequent sexual intercourse, reduced fertility, fear of taking medication, or an active underlying disease may all contribute to this [16]. Fertility in itself differs significantly depending on the underlying rheumatic disease. Female patients with inflammatory joint diseases such as RA, psoriatic arthritis (PsoA), arthritis associated with inflammatory bowel disease (CED) or axial spondylarthritis (SpA) often have an increased time to pregnancy (TTP). One quarter of individuals with RA took longer than 12 months to conceive, in contrast to 15.6 percent in the healthy control group [17]. Studies concerning fertility in women with SLE have yielded varying results [18]. The administration of cytotoxic agents, such as cyclophosphamide, in the therapy of SLE reduces ovarian reserve and thus might shorten the period of reproductive capability. An active disease during periconception may also negatively affect fertility [19]. For these reasons, fertility awareness is of utmost importance.

#### 2.2.2. Fertility Awareness

Fertility indices are at their best between the ages of 20–35, after which the decline in the number of antral follicles and quality of eggs can be observed [20]. Age, hormonal levels of follicle-stimulating hormone (FSH), oestradiol or inhibin B at an early follicular phase, the measurement of cycle-independent antimüllerian hormone (AMH) levels, as well as ultrasound markers such as antral follicle count (AFC) enable a rough estimation of the fertility potential. Inhibin B and AMHs are produced by small ovarian follicles and are therefore direct measures of the follicular pool. The diminished inhibin B secretion with increasing age or due to lower ovarian reserve results in a reduction of central negative feedback, leading to elevated pituitary secretion and higher levels of FSH in the late luteal and early follicular phases. These hormone tests are employed as possible estimative markers for assessing ovarian reserve. AMH levels and AFCs are currently the simplest, most sensitive, and specific measures of ovarian reserve. However, the EAGER trial including 1202 women showed that women with AMH values less than 1 ng/mL had similar pregnancy rates after 12 cycles of attempting to conceive as women with normal AMH values after adjustment for age [21]. Thus, the AMH level is still a poor predictor of reproductive potential measured by fecundability (the probability of conceiving in a given menstrual cycle), cumulative probability of pregnancy, or incidence of infertility [22,23]. There is no explicit cutoff to define normal or diminished ovarian reserve; moreover, they seem to be poor independent predictors of reproductive potential [22]. For this reason, these markers should only be utilized within the framework of careful interpretation by a reproductive medicine specialist. Patients with lupus nephritis or neuropsychiatric SLE who are treated with intravenous cyclophosphamide (IV CYC), which has a gonadotoxic effect, might have a reduced ovarian reserve. It is widely acknowledged that the high-dose IV CYC protocol recommended by the National Institutes of Health (NIH) has gonadotoxic effects, potentially inducing early menopause in individuals with SLE. The associated risk is proportional to both the cumulative dose administered and the age of the patients undergoing this treatment. The latest Euro-Lupus regimen of low-dose intravenous cyclophosphamide (IV CYC) (cumulative dose of 3 gm) was developed to reduce gonadal toxicity [24]. Fertility protection using the GnRH agonist treatment, oocyte cryopreservation, or embryo cryopreservation should be considered before using ovarotoxic agents, such as cyclophosphamide [12,25]. Whether the use of GnRH agonists reduces blood flow in the ovaries and gonadal toxicity is controversially discussed [26].

Gynecological factors might also influence fertility by decreasing chances of conception and implantation. In cases of the clinical suspicion of oligo-anovulatory cycles, among other conditions, polycystic ovary syndrome, premature ovarian insufficiency, hyperprolactinemia, as well as thyroid diseases which might affect the function of the hypothalamus–pituitary axis, need to be ruled out. By advanced parental age, decreased ovarian reserve, compromised egg or semen quality should be evaluated. Other gynecological reasons of infertility should be also examined, such as compromised patency of the tubes, endometriosis, or factors which might directly influence implantation, such as anatomic problems (uterine malformations, distortion of the uterine cavity from fibroids, polyps), chronic endometritis, or putative immunological dysfunctions (such as MBL deficiency, disbalance of the local NK cells), which might also decrease the receptivity of the endometrium [27]. 

**Figure 3:**
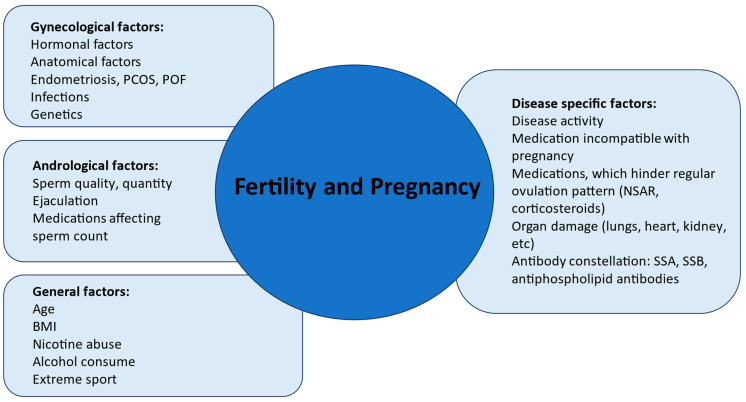
Factors affecting fertility and pregnancy in patients with SARDs. Legend: Several factors affect fertility and pregnancy course in SARDs. An interdisciplinary team should evaluate disease specific factors, which contribute to risk stratification, such as disease activity, antibody profile, organ involvement. Timing of a pregnancy should be planned at a time of disease quiescence and medication use should be adjusted to non-teratogenic therapy. Risk factors such as APL and SSA or SSB antibodies should be evaluated in order to foresee appropriate management before, during, as well as after pregnancy. Gynecological and andrological factors should be timely evaluated if time to pregnancy is over 6 months (in women older than 35) or 12 months (in individuals younger than 35) or if timing of conception is only possible in a short time window. Modifiable general risk factors should be optimized. Abbreviations: PCOS, polycystic ovary syndrome, SARD, systemic autoimmune rheumatic disease, BMI, body mass index.

Modifiable factors of infertility, such as smoking status and obesity, should be addressed and early life-style changes should be initiated (Figure 3).

Timing conception to a period of disease quiescence is a quintessential aspect of rheumatic and obstetric risk reduction [6,16]. Medication changes are often necessary during preparation for pregnancy, which can increase the risk of flare [28]. Ideally, an interdisciplinary team, comprising a rheumatologist and a gynecologist or reproductive medicine specialist, should comprehensively evaluate all factors that may influence or explicitly hinder pregnancy, and assess conditions that reduce the chances of conception. Fertility assessment and artificial reproductive techniques (ARTs) should be employed in the context of the reproductive plan, underlying disease, and ongoing medications.

Artificial reproductive techniques, such as controlled ovarian stimulation, egg retrieval, in vitro fertilization, intracytoplasmic sperm injection, and oocyte or embryo cryopreservation may play roles in facilitating optimal preparation for pregnancy. These techniques empower patients, requiring adjustments in their medications to enhance their prospects for pregnancy later on. 

#### 2.2.3. Pre-Conceptional Counselling

In a survey conducted by Chakravarty et al. only 56% of surveyed rheumatologists routinely discussed family planning with their female patients of childbearing age [29]. The most common reasons for this are that many specialists do not consider it within their competence, feel underqualified for such discussions, or find the situation uncomfortable or burdensome [30]. For this reason, multidisciplinary care including a rheumatologist and an ob/gyn specialist clearly has its advantages as it enables patients to address rheumatological problems and issues of fertility, contraception, or fertility preservation techniques until disease activity control is established and the immunomodulatory medication is compatible with the planned pregnancy. 

Individuals with SARDs are at an increased risk for maternal complications, such as disease exacerbation, hypertensive disorders of pregnancy, as well as fetal complications like preterm birth, fetal growth restriction, and neonatal heart block. Nevertheless, with the implementation of appropriate therapeutic interventions, the attainment of a successful pregnancy is mostly feasible, and risks can be minimized [30]. Pregnancy should ideally occur during a period of minimal disease activity [6,16]. Conditions such as severe renal or liver insufficiency, cardiac involvement, pulmonary hypertension, and active or severe interstitial lung disease are regarded as contraindications for pregnancy [6]. Pre-pregnancy assessments, as outlined in Table 1, are recommended to evaluate potential complications. Additionally, transitioning from medication that is incompatible with pregnancy, as detailed in Table 1, is advised prior to conception. The patient should be actively involved in treatment decisions (shared decision making). Uncertainties with regard to medication may result in compliance problems and flares.

#### 2.2.4. Pregnancy and SARD

##### How Pregnancy Affects Rheumatic Disease

Studies on SLE and RA show that disease activity before conception influences the risk of disease flare during pregnancy [19,32,33]. The quiescence of the autoimmune disease periconceptionally reduces the risk of flare and adverse obstetric outcome [19,33,34].

The PROMISSE study (Predictors of pRegnancy Outcome: bioMarker In antiphospholipid antibody Syndrome and Systemic lupus Erythematosus) from Buyon et al. showed that patients with low stable disease activity have a low risk for fetal and maternal adverse outcomes. Predictive factors for adverse outcomes are maternal flares, higher disease activity, and smaller increases in C3 levels later in pregnancy [33].

Earlier retrospective studies on RA, albeit lacking objective measurements of disease activity, reported approximately 90% of women experiencing an improvement in symptoms of their disease during pregnancy [35,36]. However, subsequent prospective studies that objectively assessed disease activity revealed a significant improvement during pregnancy in only 60% of cases, with postpartum flares reported in 47% of cases [37].

According to the PARA study from the Netherlands (Pregnancy-induced Amelioration of Rheumatoid Arthritis), antibody levels, i.e., rheumatoid factor (RF) and anti-cyclic citrullinated peptide (ACPA) do not change significantly with alterations in disease activity during and after pregnancy. Conversely, patients without these antibodies (seronegative RA) had a lower likelihood of developing a flare during pregnancy compared to patients with detectable RF and ACPA antibodies [38]. Another prospective cohort study by Förger et al. demonstrated significantly elevated levels of ACPA in patients with increased disease activity before and during pregnancy compared to women with low disease activity during pregnancy [39]. A systematic review showed a postpartum deterioration of disease activity in women with PsA [35].

##### How Rheumatic Diseases Affect Pregnancies

It has long been known that pregnancies of patients with SLE are characterized by increased risk to both mother and fetus [19,29,33]. Nevertheless, there has been a substantial improvement in pregnancy outcomes over the past six decades. In the 1960s, the incidence of stillbirths and late-term abortions in this group reached 43%, but by the year 2000, it had declined to a mere 17% [40]. In parallel, there has been a significant increase in the live birth rate among SLE patients, currently ranging from 80 to 90% [19,25,33].

A large meta-analysis including 3395 patients between 2011 and 2016 with SLE confirmed an increased risk of pregnancy-induced hypertension in pregnancy relative risk (RR 1.99), pre-eclampsia (RR 1.91), preterm labor (RR 3.05), fetal growth restriction (RR 4.44), and small for gestational age newborns (RR 1.69) [41].

According to a recent meta-analysis form 2020 examining adverse pregnancy outcomes in pregnancies involving SLE, there is a significant increase in the risk of stillbirth (risk ratio (RR) 16.49, 95% CI 2.95 to 92.13; *p* = 0.001) and fetal loss (RR 7.55, 95% CI 4.75 to 11.99; *p* = 0.00001) compared to the healthy population [42].

To identify predictive factors for adverse obstetric outcomes (APOs) in SLE patients, a comprehensive multicentric prospective study was conducted by Bouyon et al. in 2015. According to their findings, major predictors of APOs included the presence of lupus anticoagulant (LAC) (odds ratio [OR], 8.32), use of blood pressure medications (OR, 7.05), and a physician’s global assessment (PGA) score greater than 1 (OR, 4.02). Maternal flares, higher disease activity, and smaller increases in complement component 3 (C3) level later in pregnancy were also associated with APOs [33]. Given the frequent co-occurrence of antiphospholipid syndrome (APS) with autoimmune conditions, particularly SLE, the assessment of APS can aid in risk stratification and therapy in these cases. 

According to the latest systematic review and meta-analysis about RA and obstetric outcomes, increased rates of caesarean delivery (OR, 1.55), preeclampsia (OR, 1.61), and preterm delivery (OR, 1.83) were detected in pregnant individuals suffering from RA compared to control pregnant individuals. Neonatal outcomes for RA patients included higher rates of small for gestational age newborns (MD, −0.19 kg), admission to neonatal intensive care unit (OR, 1.34), and stillbirths (OR, 1.99) [43]. 

A systematic review of APOs in pregnancies with psoriasis arthritis showed a higher risk for pre-eclampsia, elective caesarean section, and preterm birth [32], while another systematic review and meta-analysis associated SpA with an increased risk of preterm birth, small for gestational age newborns, preeclampsia, and caesarean section [44].

## 3. Summary of Guidelines

While the medication recommendations in the guidelines provided by the American College of Rheumatology (ACR), British Society for Rheumatology (BSR), and European League Against Rheumatism (EULAR) for pregnant individuals with rheumatic diseases are generally similar, there are differences, which are due to available research evidence at the time of the guideline development, differences in regional practices, selected focus areas, and the composition of the expert panels. The evolution of the guidelines enables healthcare providers to find updated research data about medication use in pregnancy. Decision making should use up-to-date data and individual consideration, as documented follow-up data are scarce and, in many cases, still inconclusive.

The first guidelines were published in 2016; the expert panel composed by the EULAR addressed the question of therapy during pregnancy and breastfeeding. The primary outcome chosen was major congenital malformations in live-born children or aborted fetuses. The only secondary outcome included was miscarriages up to the 20th week of gestation. Outcomes like termination of pregnancy, preeclampsia, prematurity, fetal growth restriction (FGR), stillbirth, or postpartal infections and low Apgar score were not included as obstetric complications as they are generally incompletely documented or imprecise because of varying definitions worldwide [6]. The current guideline is under extensive revision to include the cumulated and updated evidence and address the question of new therapeutic modalities.

In 2020, the American College of Rheumatology (ACR) issued recommendations that extend beyond the guidance of medication use in patients with SARDs with or without antiphospholipid syndrome (APS). This comprehensive guideline provides evidence-based insights into critical aspects of clinical practice, specifically addressing contraception, assisted reproductive technologies (ART), fertility preservation in the context of gonadotoxic therapy, and the utilization of menopausal hormone replacement therapy (HRT) [12].

Pre-conceptional counselling is recommended to increase pregnancy success and optimize outcomes. This includes foreseeing family planning by women of reproductive age and the consideration of pregnancy-compatible, non-teratogenic, or non-gonadotoxic medications to ensure disease quiescence [12,13,14]. Unplanned pregnancy should be avoided in patients with SARDs. Due to the higher risk of disease flare and thus, the threatening of maternal health, increased risk of adverse pregnancy outcomes, and possible use of teratogenic medications, sufficient contraceptives are crucial. This guideline provides a basic understanding and suggestion of possible choices of contraceptives based on the type and activity of the rheumatic disease as well as the presence of prothrombotic antiphospholipid antibodies. The ACR recommends hormonal contraceptives or IUDs. Taking the higher risk of thrombosis into consideration, pro-thrombotic estrogen containing combined oral contraceptives are contraindicated for in APL-positive patients [12]. 

The most recent guideline, based on published evidence between 1 January 2014 to 31 December 2020, is the BSR guideline. It addresses the issue of medicament use in the pre-conceptional period and compatibility with pregnancy and breast feeding.

In summary, all recommendations state that methotrexate (MTX), mycophenolate mofetil (MMF), and CYC are teratogenic and need to be discontinued prior to conception. CYC can be considered in life-threatening conditions in the second or third trimester. According to BSR recommendations, in cases of severe (life or organ-threatening) maternal disease, CYC can be administered at any time during pregnancy. Leflunomide has been shown to be embryotoxic and teratogenic in animal studies with no corresponding malformation pattern in humans yet. However, all recommendations advise against the use of Leflunomide at the time of conception or during pregnancy due to insufficient evidence. In case of unexpected pregnancy, metabolites must be washed out with cholestyramine as soon as pregnancy is known [12]. Recommendations regarding JAK inhibitors still cannot be made due to scarce data. The voting panel of the latest guideline from 2023 could not offer recommendations regarding these drugs. It should be noted, however, that small molecules are likely to pass through the placenta and are teratogenic in animal experiments [12,13,14] (Table 1).

All of the above-mentioned medications are also not recommended during breastfeeding. 

The use of hydroxychloroquine (HCQ), Azathioprine/6-mercaptopruine, colchicine, sulfasalazine, and all TNF inhibitors is safe throughout pregnancy. To ensure low disease activity postpartum, the intake of HCQ, azathioprine, colchicine, sulfasalazine, and all TNF inhibitors is also possible during the breastfeeding period (Table 1).

According to all guidelines, hydroxychloroquine remains the medication of choice in women planning a pregnancy with rheumatic disease, especially CTD, and should be continued during pregnancy at a maximum dose of 400 mg/day. Dose restriction is based on long-term ophthalmologic safety and the fact that pregnancy reduces the reliability of weight-based dosing [45].

Concerns of the expert community were expressed regarding the European Medicines Agency’s (EMA) February 2023 recommendation to update information on hydroxychloroquine use during pregnancy [45]. The EMA’s proposal is to replace previous background information, which included data from multiple studies and a meta-analysis supporting the safety of hydroxychloroquine in pregnancy, with a single study by Huybrechts and colleagues showing a small increased risk of congenital malformations with high-dose hydroxychloroquine [46]. The revised patient leaflet suggests a small increased risk of major malformations, leading to worries about potential harm to patients and newborns. This update of the patient leaflet is not in line with the latest accumulated evidence according to the BSR Guidelines, which included and addressed the study of Huybrechts et al. in their evaluation [13]. The change in patient information may discourage hydroxychloroquine use in pregnant persons who benefit from immunomodulation, potentially causing direct harm due to disease flares [47]. Additionally, the expert panel expresses concerns about the potential indirect harm caused by patient anxiety and emotional distress. The statement of the expert community advocates for a more balanced and scientifically accurate representation of the evidence in the product characteristics, emphasizing the need for regulators to provide complete information on medication safety during pregnancy [45].

Referring to TNF inhibitors (TNFis), five agents, including etanercept (ETA), infliximab (INF), adalimumab (ADA), golimumab (GOL), and certolizumab pegol (CZP) are currently licensed. Due to their different structure, these drugs differ in their half-life and placental transfer. CZP has no or minimal placental transfer and can be continued throughout pregnancy. 

Other TNFis also may be continued throughout pregnancy to maintain disease control. However, in case of minimal risk of disease flare, withdrawal of INF, ADA, GOL, and ETA in the third trimester (ACR recommendations) [4], or INF at 20 weeks, ADA and GOL at 28 weeks, and ETA at 32 weeks (BSR recommendations) [13] should be considered (Table 1).

Other non-TNFi biologicals such as Rituximab, Interleukin-6 inhibitors, Interleukin-1 inhibitors, Interleukin-17 inhibitors, Interleukin-12/23 inhibitors have not been shown to be teratogenic. However, due to limited evidence, these biologicals should be discontinued at conception. In relation to the BSR recommendations, non-TNFi biologicals may be considered in exceptional cases with severe maternal disease and no other suitable pregnancy-compatible drug. Based on limited data, non-TNFi biologicals are compatible with breast feeding. 

The recommendation to avoid live vaccines within the first six months of infant life remains valid if treatment with biologicals was continued throughout pregnancy. 

The ACR and BSR guidelines also addressed the use of corticosteroids in pregnancy in detail. Corticosteroids like prednisolone or methylprednisolone undergo placental metabolism and retrograde transport, resulting in minimal fetal exposure [48]. Additional studies were identified (*n* = 1218 pregnancies with prednisolone and *n* = 12 pregnancies with methylprednisolone) to evaluate pregnancy outcomes. The combined data according to BSR recommendations show that birth weight and gestational duration following prednisolone exposure aligned with term pregnancies. Adverse outcomes of some studies were often linked to the underlying disease rather than steroid therapy. Additionally, limited evidence suggests the compatibility of prednisone, prednisolone, and methylprednisolone with breastfeeding, with a low transfer into breast milk. Long-term follow-up studies reported no adverse events following prednisolone exposure during pregnancy, providing reassurance regarding its safety in non-rheumatic disease pregnancies. Therefore, it is recommended to use doses <20 mg and add steroid sparing drugs if necessary [4,5].

Both ACR and BSR guidelines also include recommendations about the effect of paternal medication exposure on male fertility and teratogenicity. 

## 4. Future Needs to Improve Data and Clinical Care

### 4.1. Registries

In 2021, the EULAR Task Force published a core data set to make registries and prospective clinical studies comparable in case of inflammatory rheumatic diseases in pregnancies [49]. According to this recommendation, the first consensus-based core data set for prospective pregnancy registries in rheumatology was established. Maternal characteristics like demographics, maternal risks, such as co-morbidities and risk behavior, as well as rheumatic disease characteristics need to be documented. The course of the current pregnancy, maternal and fetal outcomes, and history of previous pregnancies should be recorded. Moreover, treatment characteristics 12 months before pregnancy, during pregnancy, and postpartum should be documented. This consensus will help to harmonize registries and collect a higher volume of comparable data on pregnant patients with rheumatic diseases. 

### 4.2. Patient Involvement in Clinical Studies

In recent years, there has been widespread advocacy for the increased participation of patient research partners (PRPs) in clinical studies to enhance awareness, compliance, and incorporate patient perspectives during conceptualization and interpretation [50,51]. Despite these efforts, the intended objectives have not been fully realized. This discrepancy is particularly evident in translational research, where the engagement of PRPs remains infrequent, and involvement levels in randomized controlled trials (RCTs) are notably low. A comprehensive literature review conducted in 2023 within the field of rheumatology revealed the absence of PRP engagement in translational research projects and a mere 50% involvement rate in RCTs [12]. Furthermore, the field of obstetrics demonstrates an even more limited inclusion of patients as research partners. Acknowledging the potential benefits, increased patient involvement in research projects would provide valuable insights into the patient perspective on diseases, fostering a more patient-centered approach with clinically relevant aims and outcomes.

### 4.3. Involvement of Pregnant Persons in Clinical Trials

The historical exclusion of pregnant persons from clinical trials, rooted in concerns about potential harm to both the pregnant person and the developing fetus, persists due to a lack of knowledge and the scarcity of trials specifically tailored to this population [52,53]. Ethical committees, tasked with safeguarding vulnerable populations, unintentionally perpetuate this cycle by enforcing exclusionary policies that impede evidence-based knowledge.

In light of the lessons learned from the recent SARS-CoV-2 pandemic, it becomes imperative to advocate for the inclusion of pregnant persons with rheumatic diseases in clinical trials. The vulnerability of specific populations, including those with autoimmune conditions, has been underscored during the pandemic. Global centers have recognized the obligation to include such vulnerable groups in clinical trials. Excluding pregnant persons with rheumatic diseases from these trials not only compromises the generalizability of study findings but also hinders their access to potential therapeutic advancements [54].

In the field of rheumatology, compelling evidence highlights the efficacy of immunomodulatory medications in controlling diseases and improving obstetric outcomes [10,12,13]. In light of this evidence, it is imperative to reevaluate the ethical justification for the ongoing exclusion of pregnant persons from clinical trials. The discourse should advance towards devising practical strategies for the careful and deliberate inclusion of pregnant persons in clinical research, especially concerning immunomodulatory drugs. Policymakers and other stakeholders must actively contribute to promoting this transformative change, fostering policies that address the unique needs of pregnant individuals while advancing our understanding of drug safety in this vulnerable population. The guidance provided by the federal Task Force on Research Specific to Pregnant Persons and Lactating Women further emphasizes the importance of including this population in research [55], reinforcing the need to extend this inclusion to pregnant persons with rheumatic diseases for the development of tailored and effective therapies.

### 4.4. Challenges and Future Directions of Improvement

The definition of adverse obstetric outcomes lacks standardization, leading to variations in research endpoints when exploring the association between rheumatic disease and obstetric outcomes. A recent systematic review investigated composite adverse outcomes in obstetric studies, identifying 156 RCTs with 181 composite outcomes related to general morbidity and mortality. Among these, 158 outcomes focused on maternal and fetal–neonatal outcomes, or both. Notably, obstetric composite outcomes, ranging from two to sixteen components, exhibited significant differences in severity, frequency, and alignment with the study’s primary objective. Some components were unrelated to the study’s focus [56]. The increasing use of composite outcomes in obstetric RCTs lacks consistency in component selection and measurement methods, challenging the comparison and utilization of results in guideline development.

There is a clear need for a better definition of maternal and fetal adverse outcomes, along with improved standardization of disease characterization (including disease activity measures, time points in pregnancy, and adaptation of activity scores during pregnancy) in SARDs during pregnancy.

Given the EULAR Task Force’s efforts to harmonize registries, there is a hopeful expectation for higher sample sizes to address whether medication during pregnancy potentially harms the fetus. Uncertainties in recommendations stem from small sample sizes, as demonstrated by a theoretical power analysis for the incidence of congenital malformations associated with immunomodulatory medications during pregnancy. What follows is a power calculation considering a background risk of 4% in the healthy population without medication use (estimated between 3 and 5% according to the literature). Assuming a 2% absolute increase in the risk of congenital malformations due to the medication corresponds to an odds ratio of roughly 1.5. Under the standard assumptions of a significance level of 0.05 and a desired power of 0.8, we calculated that we would need 1863 patients per group, or 3726 in total, in the case of using a two-sided Χ2 test, in order to actually be able to observe a statistically significant result [57,58].

The small number of exposed cases in the current registries, lack of long-term follow-up, as well as varying outcome parameters, multiple medication use, and the confounding role of the underlying disease poses an enormous challenge on the recommendation development as well as on counselling in clinical practice.

## 5. Recommendations for Practice

**Figure 4:**
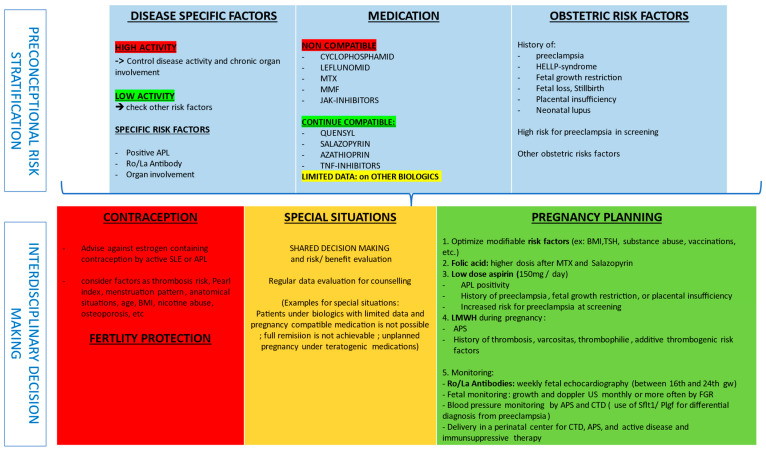
Pre-conceptional decision making and risk stratification. Legend: Patients with SARDs should receive interdisciplinary pre-pregnancy counselling to assess disease activity, medication safety, stratification of specific risk factors such as APL, organ involvement, obstetric history, and assessment of reproductive potential in order to optimize pre-pregnancy care. Modifiable risk factors, such as BMI, vaccination, smoking, and control of other comorbidities should be optimized. Abbreviations: APS, antiphospholipid syndrome; CTD, connective tissue disorder; LMWH, low molecular weight heparin; HELLP, hemolysis, elevated liver enzymes and low platelets; MTX, methotrexate; MMF, mycophenolate mofetil; SLE, systemic lupus erythematosus; TNF, tumor necrosis factor; TSH, thyroid stimulating hormone.

Patients with SARDs should receive interdisciplinary pre-pregnancy counselling to assess disease activity, medications, stratification of specific risk factors such as APL, organ involvement, obstetric history, and the assessment of reproductive potential in order to optimize pre-pregnancy care. Modifiable risk factors, such as weight, vaccination, smoking, and control of other comorbidities, should be optimized (Figure 4).

Specialist counselling should include an assessment of disease activity in each trimester as well as obstetric monitoring tailored to possible risk factors during pregnancy. As disease flare postpartum is common, disease activity assessment and monitoring are recommended until four months after delivery. Counselling about medication use during breastfeeding should support participative decision making of the patient.

## 6. Inclusivity

When possible, we used “individuals” or “person” instead of “women” in this review to demonstrate inclusivity for all gender identities.

## Figures and Tables

**Table 1:** Summary of recommendations in SARDs concerning medications in pregnancy.

Substance	Pre-Conception	Pregnancy	Lactation	Recommendation/Comment
Nonsteroidal antiinflammatory drugs				Discontinue if time to pregnancy longer. Possible in the first and second trimester, discontinue in the 30–32nd week of pregnancy at the latest. Ibuprofen should be preferred during breastfeeding.
Prednisone				Taper to the minimum effective dose (<20 mg). Add pregnancy-compatible immunosuppressants, if neccesary. After a dose of >20 mg, delay breastfeeding for 4 h.
Conventional medications				
Azathioprine/6-mercaptopurine				
Hydroxychloroquine				Dose of <400 mg/day
Colchicine				
Sulfasalazine				Increased folic acid substitution (5 mg per day) is recommended up to 12 weeks of gestation.
Cyclophosphamide	(3 months)			Exception for life/organ-threatening diseases in the 2nd and 3rd trimester (after embryonic organ formation is complete).
Methotrexate	(1–3 months)			For women treated with MTX within one month prior to conception, increased folic acid supplementation (5 mg per day) is recommended up to 12 weeks of gestation.
Leflunomide	(24 months)			Teratogenic in animal studies, human data not sufficient for a recommendation. Half-life 2 years, in case of desire to have children or unplanned pregnancy cholestyramine washout (8 g three times a day for 11 days) is recommended.
Mycophenolate mofetil	(1.5 months)			
Cyclosporin A				Monitor blood pressure
Tacrolimus				Monitor blood pressure
Targeted synthetic DMARDs				
JAK-inhibitors	(2 weeks)			Unable to make a recommendation due to insufficient data; small molecular size suggests transfer across the placenta and into breast milk
Tumor necrosis factor inhibitors				
Adalimumab				Evaluate continuation in the 28th week of pregnancy. Live vaccinations of the infant should be postponed until 6 months of age, if given in late pregnancy.
Infliximab				Evaluate continuation in the 20th week of pregnancy. Live vaccinations of the infant should be postponed until 6 months of age, if given in late pregnancy.
Etanercept				Evaluate continuation in the 32th week of pregnancy. Live vaccinations of the infant should be postponed until 6 months of age, if given in late pregnancy.
Certolizumab				Low/no diaplacental transport. Requires no change to the vaccination schedule for infants.
Golimumab				Evaluate continuation in the 28th week of pregnancy. Live vaccinations of the infant should be postponed until 6 months of age, if given in late pregnancy.
Other biologics				
IL-1-inhibitors				Limited evidence has not shown that “other biologics” are teratogenic. However, due to insufficient evidence stopping the drug at conception is recommended. They may be considered to manage severe maternal disease in pregnancy, if no other pregnancy-compatible drug is effective. Based on limited evidence breastfeeding is possible.
Abatacept				
Rituximab				
IL-6-inhibitors				
Belimumab				
IL-17-inhibitors				
IL-12/23-inhibitors				
	substance may be applied			
	data is insufficient for substance recommendation			
	substance application is not recommended			

Legend: Modified after [31]. In summary, avoid methotrexate, mycophenolate mofetil, and cyclophosphamide during pregnancy. Leflunomide is discouraged due to potential risks, and if used, metabolites should be eliminated upon pregnancy confirmation. The use of hydroxychloroquine (HCQ), Azathioprine/6-mercaptopruine, colchicine, sulfasalazine, and all TNF inhibitors is safe throughout pregnancy and during breastfeeding. Limited data and placental transfer of JAK inhibitors necessitate caution. Non-TNFi biologicals (e.g., Rituximab, IL-6/IL-1/IL-17/IL-12/23 inhibitors) have not shown teratogenicity. Limited evidence suggests discontinuation at conception. BSR allows non-TNFi use in severe cases with no pregnancy-compatible alternative. Limited data support compatibility with breastfeeding.

## Abbreviations

ACPA	Anti-Citrullinated Protein Antibodies
ACR	American College of Rheumatology
AFC	Antral Follicle Count
AMH	Anti-Mullerian Hormone
APO	Adverse Pregnancy Outcomes
aPL	Antiphospholipid antibodies
APS	Antiphospholipid Syndrome
ART	Assisted Reproductive Technology
BSR	British Society for Rheumatology
CED	Inflammatory bowel disease
CI	Confidence Interval
CTD	Connective Tissue Disease
CYC	cyclophosphamide
EMA	European Medicines Agency
EULAR	European League Against Rheumatism
FGR	Fetal Growth Restriction
FSH	Follicle-Stimulating Hormone
HCQ	Hydroxychloroquine
HRT	Hormone replacement therapy
IJD	Inflammatory joint disease
KG	Kilogram
LAC	Lupus anticoagulant
MD	Mean Difference
MMF	Mycophenolate mofetil
MTX	Methotrexate
OR	Odds Ratio
PARA	Pregnancy induced Amelioration of Rheumatoid Arthritis
PGA	Physician’s Global Assessment
PRP	Patient research Partners
PsA	Psoriatic Arthritis
RA	Rheumatoid Arthritis
RCT	Randomized controlled trials
RF	Rheumatoid Factor
RR	Risk Ratio
SARD	Systemic Autoimmune Rheumatic Diseases
SARS-CoV-2	Severe Acute Respiratory Syndrome Corona Virus Disease 2019
SLE	Systemic Lupus Erythematosus
SpA	Spondyloarthritis
TNF	Tumor necrosis factor
